# Worse Renal Disease in Postmenopausal F2[Dahl S x R]-Intercross Rats: Detection of Novel QTLs Affecting Hypertensive Kidney Disease

**DOI:** 10.1371/journal.pone.0056096

**Published:** 2013-02-05

**Authors:** Victoria L. M. Herrera, Khristine A. Pasion, Ann Marie Moran, Nelson Ruiz-Opazo

**Affiliations:** Section of Cardiovascular Medicine, Department of Medicine, Boston University School of Medicine, Boston, Massachusetts, United States of America; Fondazione IRCCS Ospedale Maggiore Policlinico & Fondazione D′Amico per la Ricerca sulle Malattie Renali, Italy

## Abstract

The prevalence of hypertension increases after menopause with 75% of postmenopausal women developing hypertension in the United States, along with hypertensive end organ diseases. While human and animal model studies have indicated a protective role for estrogen against cardiovascular disease and glomerulosclerosis, clinical studies of hormone replacement therapy in postmenopausal women have shown polar results with some improvement in hypertension but worsening of hypertensive kidney disease, or no effect at all. These observations suggest that the pathogenesis of postmenopausal hypertension and its target organ complications is more complex than projected, and that loss of endogenous estrogens induces epigenetic changes that alter genetic susceptibility to end-organ complications per se resulting in pathogenetic mechanisms beyond correction by hormone replacement. We studied postmenopausal-induced changes in renal disease and performed a total genome scan for quantitative trait loci (QTLs) affecting kidney disease in postmenopausal 16m-old F2[Dahl S x R]-intercross female rats. We used glomerular injury score (*GIS*) as quantitative trait. We compared QTLs amongst premenopausal, ovariectomized and postmenopausal F2[Dahl S x R]-intercross rats using identical phenotype characterization. Postmenopausal F2[Dahl S x R]-intercross rats exhibited increased hypertensive glomerulosclerosis (P<0.01) and equivalent levels of kidney disease when compared to premenopausal and ovariectomized F2[Dahl S x R]-intercross rats respectively. We detected three significant to highly significant GIS-QTLs (*GIS-pm1* on chromosome 4, LOD 3.54; *GIS-pm2* on chromosome 3, LOD 2.72; *GIS-pm3* on chromosome 5, LOD 2.37) and two suggestive GIS-QTLs (*GIS-pm4* on chromosome 2, LOD 1.70; *GIS-pm5* on chromosome 7, LOD 1.28), all of which were unique to this postmenopausal population. Detection of increased renal disease phenotype in postmenopausal and ovariectomized subjects suggests a protective role of ovarian hormones. Furthermore, the detection of distinct *GIS*-QTLs in postmenopausal intercross female rats suggests that distinct genetic mechanisms underlie hypertensive glomerulosclerosis in premenopausal and postmenopausal states.

## Introduction

In general, hypertensive kidney disease is less common in women compared to men [1]–[5], however this gender protective effect diminishes and tends to disappear with the onset of menopause [6] leading to the accepted paradigm that hormone replacement in postmenopausal women would prevent hypertension and its end-organ complications such as hypertensive kidney disease. However, cumulative data report polar results regarding the benefits from hormone replacement therapy for hypertension [7] as well as for its end-organ complications with some studies reporting improvement in kidney function [8]–[10] while other studies detecting either no change [11] or worsening of postmenopausal kidney function in postmenopausal women [12]. These discordant results have been attributed to several factors such as the use of distinct study populations [8], [9], [11], the type of hormone replacement therapy (estrogen or progestin), and/or differences in dosing or route of delivery [9], [11]. Alternatively, barring technical errors, discordant observations on the efficacy of hormone replacement therapy for postmenopausal hypertension raises the hypothesis that the pathogenesis of hypertensive kidney disease is far more complex than projected and is currently poorly understood. Differential pathogenic mechanisms could underlie postmenopausal hypertension and its end-organ diseases thus explaining differential response to the same therapy, or that hormone replacement could simply be non-protective, if not detrimental to post-menopausal hypertensive kidney disease or renal function in general.

Given that some patients have worsening of kidney function with hormone replacement therapy [12], it is important to determine the phenotype of hypertensive kidney disease in a postmenopausal F2[Dahl S x R]-intercross rat cohort which has demonstrated increased salt-sensitive hypertension in postmenopausal rats far greater than the mean blood pressure levels reached in both premenopausal female rats and premenopausal ovariectomized female rats [13], and wherein experimental environmental and genetic confounders are minimized if not eliminated. Furthermore, given that distinct genetic factors, or quantitative trait loci (QTLs), underlie premenopausal hypertension [13], [14] as well as in premenopausal ovariectomized female rats [15], it is important to determine whether distinct QTLs also underlie postmenopausal hypertensive kidney disease. Identification of distinct QTLs for postmenopausal renal disease will elucidate that premenopausal and postmenopausal kidney disease has distinct pathogenic mechanisms. This is important to dissect and elucidate as prerequisites to the identification of novel approaches to prevent or treat postmenopausal kidney disease.

In order to investigate potential differences in genetic mechanisms underlying premenopausal and postmenopausal susceptibility to hypertensive renal disease, we performed a total genome scan for QTLs affecting glomerular injury score (GIS) as a quantitative measure of hypertensive nephrosclerosis [16] using an F2 (Dahl S x R)-intercross postmenopausal female rat population characterized for glomerular injury scores. Results were then compared to phenotype characteristics and genome scan results obtained in premenopausal [14] and premenopausal ovariectomized F2 [Dahl S x R]-intercross female rats [15]. Data demonstrate that distinct QTLs underlie postmenopausal hypertensive renal disease in contrast to premenopausal kidney disease.

## Results

### Increased hypertensive nephrosclerosis in postmenopausal rats

**Figure 1:**
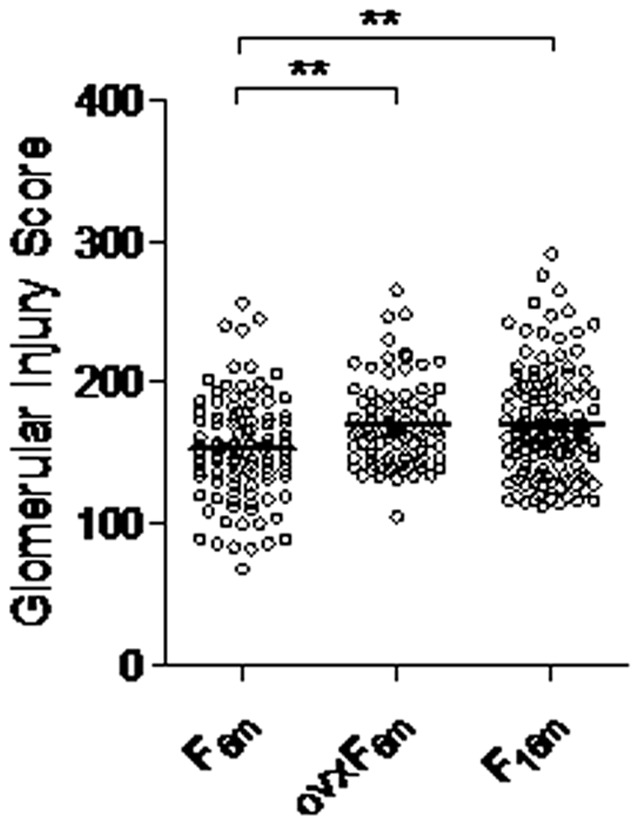
Distribution of GIS in premenopausal, ovariectomized and postmenopausal F2 (Dahl S x R)-intercross cohorts. Distribution of GIS in premenopausal F2 (F_6m_, n = 102) female hybrids at 6 months of age (8% NaCl challenge begun at 3 months of age); ovariectomized female F2 (ovxF_6m_, n = 116) hybrids at 6 months of age (8% NaCl challenge begun at 3 months of age) and postmenopausal F2 (F_16m_, n = 130) female hybrids at 16 months of age (8% NaCl challenge begun at 14 months of age). Means are shown as horizontal lines. ^**^ *P*<0.01 (one-way ANOVA followed by Holm-Sidak test for multiple comparisons).

To determine whether worse hypertension observed in postmenopausal rats [13] is also associated with increased end-organ disease or the opposite as observed in some clinical studies [12], we analyzed established quantitative measures of hypertensive nephrosclerosis, glomerular injury scores (GIS) [16] and compared GIS measures among postmenopausal F2[Dahl S x R]-intercross rats, premenopausal ovariectomized F2[Dahl S x R]-intercross rats [15], and premenopausal F2[Dahl S x R]-intercross rats [14]. As shown in Figure 1, glomerular injury scores were increased significantly in postmenopausal and premenopausal ovariectomized F2[Dahl S x R]-intercross rats when compared with premenopausal female rats (P<0.01). These results indicate concordance of hypertension and renal disease susceptibility in postmenopausal F2[Dahl S x R]intercross-rats showing increased susceptibility to salt-sensitive hypertension [13] and hypertensive nephrosclerosis in postmenopausal state.

**Figure 2:**
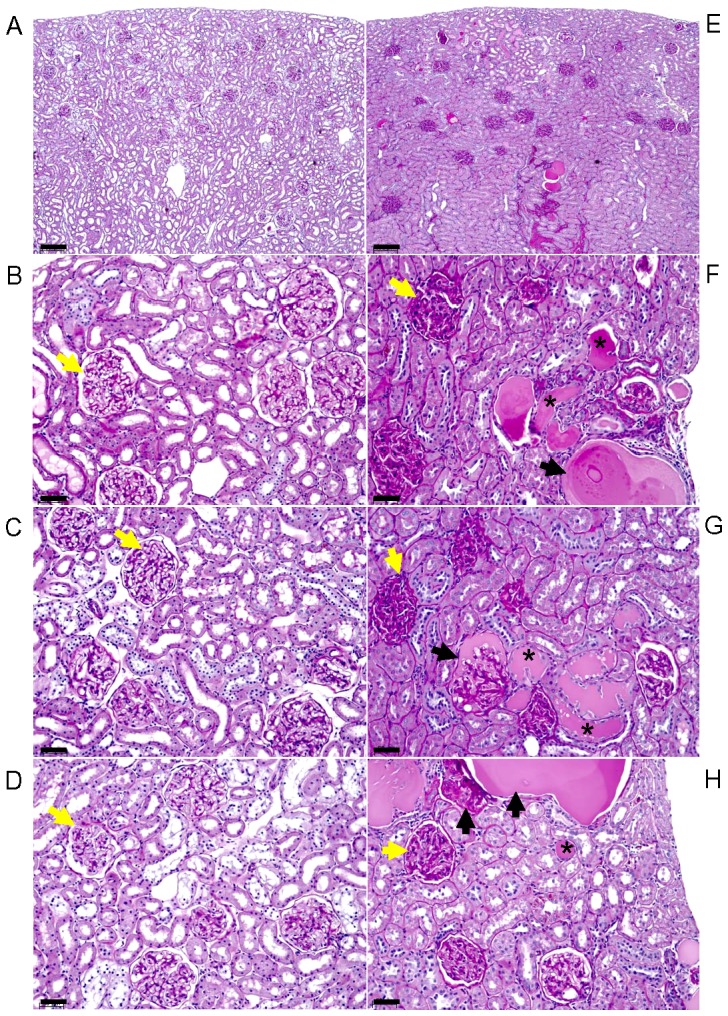
Representative Periodic Acid Schiff (PAS)-stained photomicrographs of rat kidneys with low (≤115) and high (≥265) glomerular injury scores (GIS). A. Low magnification view of representative rat kidney section with low GIS, and (B–D) high magnification view of three representative rat kidney sections with low GIS scores demonstrate mild PAS-staining (magenta) of glomerular mesangial matrix and basement membranes, minimal PAS-staining of tubular basement membranes and tubular casts, and no glomerular sclerosis. In contrast, E, low magnification view of representative rat kidney section with high GIS, and (F–H) high magnification view of three representative rat kidney sections with high GIS show increased number of PAS-stained glomeruli with thickened mesangial matrix and basement membranes, glomerular sclerosis, tubular casts and PAS-stained thickened tubular basement membranes. Yellow <$>\raster(80%)="rg1"<$>, glomeruli, <$>\raster(80%)="rg1"<$> sclerotic glomeruli, *, tubular casts, bar = 200 micron (A, E), 50 microns (B–D, E–H).

In order to demonstrate the robust spectrum of the hypertensive kidney disease phenotype measured by GIS, histological photomicrographs images are presented representing the tail-ends of the spectrum of GIS scores (Figure 2). Histological review of representative rat kidney sections with low GIS (≤115) shows that low GIS is associated overall with less glomerulosclerosis, less thickening of glomerular mesangial matrix and basement membrane, less number of tubular casts and tubular basement membrane thickening (Figure 2 A–D). In marked contrast, representative kidney sections with high GIS scores (≥265) kidneys show glomerulosclerosis, thickening of glomerular mesangial matrix and basement membrane, as well as thickening of renal tubular basement membranes and more tubular casts (Figure 2 E–H). The analysis of representative histological images of low (≤115) vs high (≥265) GIS values reveal the concordance of histopathological severity of hypertensive kidney disease with GIS, thus corroborating the robust phenotype measure.

### Distinct GIS QTLs in postmenopausal F2[R x S]-Intercross Female Rats

**Figure 3:**
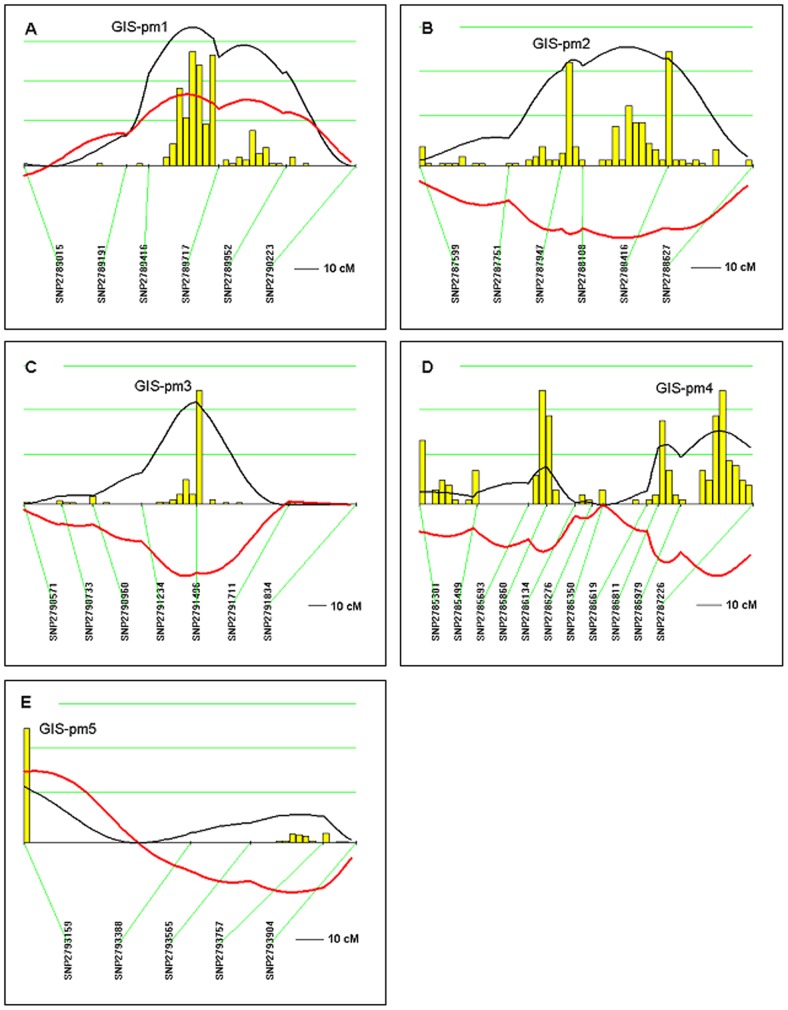
QTLs for glomerular injury score (GIS) in postmenopausal F2 (Dahl S x R)-intercross female rats. Chromosomes were analyzed by interval mapping with bootstrap resampling method to estimate a confidence interval (QTXb19 Map Manager): Panel A, chromosome 4 (*GIS-pm1*); B, chromosome 3 (*GIS-pm2*); C, chromosome 5 (*GIS-pm3*); D, chromosome 2 (*GIS-pm4*) and E, chromosome 7 (*GIS-pm5*). Yellow histograms represent the bootstrap-based confidence intervals for the detected QTLs. Orientation of chromosomes: left → right starting from lowest Mbp. Horizontal green lines [] mark LOD values for significance of linkage, from top to bottom: highly significant LOD≥3.17; significant LOD≥2.17; suggestive LOD≥1.15; LOD [—]; regression coefficient [**—**].

One hundred and thirty postmenopausal F2 female hybrids were genotyped at 100 markers informative for Dahl S and Dahl R strains. Using dominant and recessive models, we detected five GIS-QTLs not previously reported in premenopausal [14] and premenopausal ovariectomized [15] females (Table 1, Figure 3). One with highly significant linkage on chromosome 4, *GIS-pm1* (Figure 3A, LOD = 3.54), two with significant linkage on chromosome 3 *GIS-pm2* (Figure 3B, LOD = 2.72) and on chromosome 5 *GIS-pm3* (Figure 3C, LOD = 2.37), and two with suggestive linkage on chromosome 2 *GIS-pm4* (Figure 3D, LOD = 1.70) and on chromosome 7 *GIS-pm5* (Figure 3E, LOD = 1.28). We note that in *GIS-pm1, GIS-pm4 and GIS-pm5*, the S allele decreases susceptibility to renal disease, thereby corroborating distinct pathogenic mechanisms involved in susceptibility to glomerulosclerosis in premenopausal and postmenopausal states.

**Table 1:** QTLs for glomerular injury score in postmenopausal F2 (Dahl S x R) intercross female rats.

QTL	Model	Rat-location	LOD	%
GIS-pm1	Recessive	Chr4: 74–112 Mbp	3.54 (HS)	12 (↓)
GIS-pm2	Dominant	Chr3: 80–120 Mbp	2.72 (S)	9 (↑)
GIS-pm3	Dominant	Chr5: 125–145 Mbp	2.37 (S)	8 (↑)
GIS-pm4	Dominant	Chr2: 215–235 Mbp	1.70 (Sug)	6 (↓)
GIS-pm5	Recessive	Chr7: 15–35 Mbp	1.28 (Sug)	5 (↓)

QTL, quantitative trait locus; GIS, glomerular injury score; Chr, chromosome; % , the amount in % of total trait variance that would be explained by a QTL at these loci; Mbp, mega-base pair; LOD, logarithm of the odds score derived from the likelihood ratio statistic using a factor of 4.6; ↑, S-allele increases trait; ↓, S-allele decreases trait. Significance determined from 2000 permutations on data set: LOD 3.17 highly significant (HS); LOD 2.17 significant (S); LOD 1.15 suggestive (Sug).

### Interacting Loci Detected in postmenopausal F2 Female Cohort

Our analysis also reveals a number of gene-gene interactions in the postmenopausal female F2-intercross cohort for GIS that surpassed the threshold criteria for interaction (Table 2). In contrast, a single interacting-locus was detected in the premenopausal female F2[Dahl S x R]intercross-rat cohort affecting glomerular injury score that fulfilled criteria for gene interaction [14].

**Table 2:** Gene interaction analysis of GIS in postmenopausal F2 (Dahl S x R) intercross female rats.

ChrA	QTLA	Locus A (Mbp)	ChrB	QTLB	Locus B (Mbp)	T	I	Main A	Main B
4	GIS-pm1	SNP2789416 (73.7)	3	GIS-pm2	SNP2788416 (120.0)	12.9	7.8	2.4	2.3
4	GIS-pm1	SNP2789952 (140.7)	5	GIS-pm3	SNP2791496 (134.9)	10.7	5.5	2.4	2.8
2	GIS-pm4	SNP2786811 (196.3)	5	GIS-pm3	SNP2791496 (134.9)	10.6	7.0	1.4	2.3
2	GIS-pm6	SNP2785860 (77.5)	3	GIS-pm2	SNP2787751 (41.2)	10.5	8.3	0.9	1.3
3	GIS-pm2	SNP2787947 (60.4)	5	GIS-pm3	SNP2791496 (134.9)	10.0	5.8	2.3	2.3

Only interactive loci that exhibited a LOD>9.2 (*P*<10^−6^) for the total effect and a LOD>2.7 (*P*<0.01) for the interactive effect are presented. *Main A* and *Main B* refer to the specific LOD for *A* and *B* main (single locus) effects respectively. *T*, LOD for total effect; *I*, LOD for the interaction; *GIS*, glomerular injury score.

## Discussion

Premenopausal females are less susceptible than men to hypertensive renal disease [17] and heart disease [18]. However, after menopause the incidence of chronic renal disease in women increases suggesting that the loss of sex hormones contributes to the development and progression of kidney disease [6]. This is supported by some experimental data. In studies using ovariectomized Dahl-S rats as an animal model of surgically-induced postmenopausal salt-sensitive hypertension, 17β-estradiol attenuated age-related renal dysfunction [19], [20]. However, studies of hormone replacement therapy in aging women are controversial, with some studies showing improvement [8]–[10] and others showing loss of kidney function [12].

Our studies in premenopausal, premenopausal-ovariectomized and spontaneously postmenopausal female rats clearly show that hypertensive kidney disease or nephrosclerosis, measured as glomerular injury score, is more severe in postmenopausal and ovariectomized rats compared with premenopausal rats concordant with higher blood pressure levels detected in postmenopausal rats when compared with premenopausal rats [13]. These results confirm differential pathogenesis of hypertension and its end-organ diseases in premenopausal and post menopausal states, as well as corroborate the role of ovarian hormones in postmenopausal hypertension–protective role(s) for blood pressure and for hypertensive renal disease. These observations are concordant with the studies reporting that estrogen/progestin replacement therapy improved renal function [8]–[10].

The identification of five distinct QTLs affecting renal disease in postmenopausal rats but which were not detected in premenopausal [14] and premenopausal ovariectomized F2[Dahl S x R]-intercross rats [15] demonstrate distinct pathogenetic mechanisms involved in development and progression of kidney disease in postmenopausal rats. These observations also indicate complex roles of sex hormones in postmenopausal female heath, and that given the known complexity of aging mechanisms, hormone replacement for postmenopausal hypertension and its complications would likely not suffice for projected restorative efficacy.

In three out five GIS-QTLs detected in postmenopausal rats the S allele decreased susceptibility to hypertensive glomerulosclerosis. In contrast, in both premenopausal [14] and ovariectomized [15] cohorts the S allele increased susceptibility to kidney disease. Analysis of reported QTLs affecting glomerulosclerosis in other Dahl S intercrosses using different normotensive strains revealed that *GIS-pm2* and *GIS-pm4* chromosomal regions overlapped with QTLs affecting glomerular injury in a premenopausal female F2[Dahl S x Brown Norway]intercross rat cohort [21], however the directionality of the *GIS-pm4* S allele effect on renal disease differed with the S allele decreasing susceptibility in our postmenopausal cohort and increasing susceptibility in the premenopausal F2[Dahl S x Brown Norway]intercross rat population [21]. This suggests that the gene underlying *GIS-pm2* might affect glomerulosclerosis independently of environment and age, in contrast to the gene accounting for *GIS-pm4* which appears to affect renal disease in an environment and age-dependent manner. Identification of corresponding gene variants will be necessary to verify this hypothesis.

Candidate gene analysis encompassing ±15 Mbp from the peaks of GIS QTLs detected with significant and highly significant linkage revealed several candidate genes. For *GIS-pm1* on chromosome 4 Adcyap1r1 (adenylate cyclase activating polypeptide 1 receptor 1) gene is located at Chr4-84.2 Mbp. For *GIS-pm2* QTL peak on chromosome 3 Avp (arginine vasopressin) gene localizes to Chr3-118.2 Mbp and for *GIS-pm3* the CYP4A gene cluster (Chr5-135.5 Mbp) and Edn2 (endothelin 2) at chr5-140.7 Mbp are possible candidates. Interestingly, Adcyap1r1 (also known as PAC1 receptor) and its ligand (PACAP) are present in kidney, mainly on tubular epithelial cells [22]. The PAC1R/PACAP system has been associated with tubuloprotective effects in the kidney [23]–[25]. Avp mapping to *GIS-pm2* is a potential candidate based on its well known relationship to chronic kidney disease [26]. On the other hand, the CYP4A gene cluster has been shown to affect renal injury in male congenic Dhal S rats introgressed with the chromosome 5 CYP4A region of Lewis rats [27] and Edn2 has been implicated in feline naturally occurring renal failure [28] and in development of glomerulosclerosis in transgenic rats expressing the human Edn2 [29], thus both genes could be candidates that might underlie the effect of *GIS-pm3* on hypertensive nephrosclerosis as represented in GIS measures. 

In conclusion, our study demonstrates the involvement of distinct genetic mechanisms in premenopausal and postmenopausal hypertensive renal disease, and confirms the paradigm that differential genetic mechanisms underlie hypertension and its different target-organ complications in both premenopausal and postmenopausal states. These observations imply that treating hypertension resulting in the lowering of blood pressure does not necessarily address hypertensive target organ complications since the latter have distinct genetic mechanisms as demonstrated by different QTLs. The elucidation of distinct genetic mechanisms in premenopausal and postmenopausal hypertension as well as in hypertensive end organ diseases establishes a primary etiological framework for novel prevention and intervention strategies that needs to be studied further.

Altogether, these data mandate that the study of hypertension genes requires stage specific analyses for female hypertensives. Given that current anti-hypertensive therapies and prevention strategies have not eradicated hypertension nor adequately addressed postmenopausal hypertension, studies of postmenopausal hypertension and end-organ diseases need to be prioritized, if not mandatory, especially since hypertension remains a major risk factor for heart disease, kidney disease and stroke, which combine to be the number-one causes of mortality and morbidity in females.

## Materials and Methods

### Ethics Statement

This study was performed in strict accordance with the recommendations in the Guide for the Care and Use of Laboratory Animals of the National Institutes of Health. The protocol was approved by the Committee on the Ethics of Animal Experiments of Boston University School of Medicine (Permit Number: AN-14966).

### Genetic Crosses

Inbred Dahl S/jrHsd and Dahl R/jrHsd rats were obtained from Harlan (Indianapolis, Indiana). Parental strains (Dahl R/jrHsd female x Dahl S/jrHsd male) were crossed to produce F1 progeny. The F2 subjects were derived from brother-to-sister mating of F1 hybrids to produce the F2 female (n = 130) segregating population.

### Ascertainment of postmenopausal status

In order to ascertain postmenopausal status of the female F2 hybrids daily vaginal smears were performed on females to determine the stage of their estrous cycle (diestrous, proestrous or estrous) essentially as described [30], [31]. Female hybrids bred for phenotypic characterization were subjected to vaginal smears commencing at 12 months of age until 14 months of age. Cessation of cycling in the female hybrids was defined as continuous estrous for 4 weeks [30]. The postmenopausal F2 population showed that >99% of females stopped cycling by 14 months of age.

### Phenotypic Characterization

Female subjects were maintained on a low salt (0.008 % NaCl) diet until high salt (8 % NaCl) challenge began at 14 months of age to avoid raises in blood pressure prior the high salt challenge. Females were fed a high salt (8 % NaCl) diet for 8 weeks. At 16 months of age animals were sacrificed and kidneys were immediately removed, fixed in 4% paraformaldehyde, processed for histology and renal pathology quantified as described [14], [32]. All glomeruli (on average n = 209) in one frontal renal section were analyzed in a blind manner for degree of glomerulosclerosis and mesangial matrix expansion. Glomerulosclerosis was defined as disappearance of cellular elements from the tuft, collapse of capillary lumen, and folding of the glomerular basement membrane with entrapment of amorphous material. Mesangial matrix expansion was defined by the presence of increased amounts of PAS-positive material in the mesangial region. PAS-stained slides were reviewed using a Zeiss Axioskop microscope and digital photomicroscopy performed using identical settings to validate visual comparative analysis. Cortical glomeruli and tubules were analyzed at low (50x) and high magnification (200x). Renal pathology grade I, 0–25% involvement of glomerulus with pathology; II, 26–50% involvement; III, 51–75% involvement; IV, 76–100% involvement. For premenopausal and postmenopausal cohorts comparison the extent of injury for each renal section was calculated as the glomerular injury score (GIS) = (1 x % grade I)+(2 x % grade II)+(3 x % grade III)+(4 x % grade IV), increasing with worse injury represented by glomerulosclerosis and mesangial matrix expansion [14], [32]. For linkage analysis we used [% grade III+% grade IV] as quantitative trait because gave the most robust results on QTL analysis.

### Intercross Linkage Analysis

Distributions were analyzed for normality; data transformations were done and datasets that passed Kolmogorov-Smirnov normality testing (SigmaStat) were used for linkage analysis. QTL analysis was performed using glomerular injury score (GIS) defined as [% grade III+% grade IV] as quantitative trait. In our postmenopausal female F2 intercross population, previously characterized for blood pressure [13], renal disease (GIS) showed no significant relationship with systolic blood pressure upon Pearson Product Moment Correlation analysis (GIS-SBP Correlation Coefficient  = 0.149, P = 0.09), thus QTL analysis for GIS was performed without adjustment for BP. Linkage maps, marker regression and composite interval mapping were done with the Map Manager QTXb19 (MMQTXb19) program for windows [33] which generates a likelihood ratio statistic (LRS) as a measure of the significance of a possible QTL. Genetic distances were calculated using Kosambi mapping function (genetic distances are expressed in centiMorgan, cM). Critical significance values (LRS values) for interval mapping were determined by a permutation test (2000 permutations at all loci tested) on our postmenopausal female cohort using Kosambi mapping function and a constrained (dominant or recessive) regression model. Values for suggestive linkage LRS = 5.3 (LOD 1.15), for significant linkage LRS = 10.0 (LOD 2.17) and for highly significant linkage LRS = 14.6 (LOD 3.17). LRS 4.6 delineates LOD 1-support interval. Confidence interval for a QTL location was estimated by bootstrap resampling method wherein histogram single peak delineates the QTL and peak widths define confidence interval for the QTL. Histograms which show more than one peak warn that the position for the QTL is not well defined or that there may be multiple linked QTLs (QTX Map Manager). We also performed interaction analysis using the Map Manager QTXb19 program applying a two-stage test paradigm for determination of interaction in which the pair of loci must pass two tests in order to be reported as having a significant interaction effect. First, the significance of the total effect of the two loci must be <0.000001 and second, the pairs of loci must exhibit a *P* value <0.01 for the interaction effect.

### Genotyping

SNP genotyping was carried out on an Applied Biosystems 7900 Real-Time PCR System. SNPs (n = 97) and SSLP markers (n = 3) were selected from the RGD SNP database. SNP assays (TaqMan assays) were procured from Applied Biosystems and were validated in our laboratory.

### Statistical Analysis

Analysis of phenotypes in F2 female cohorts was done by one-way ANOVA followed by all pairwise multiple comparisons using Holm-Sidak test. The statistical analysis was performed using the software SigmaPlot for Windows Version 11.0, Build 11.2.0.5 (2008 Systat Software, Inc.).
